# Cesium tolerance is enhanced by a chemical which binds to BETA-GLUCOSIDASE 23 in *Arabidopsis thaliana*

**DOI:** 10.1038/s41598-021-00564-4

**Published:** 2021-10-26

**Authors:** Ju Yeon Moon, Eri Adams, Takae Miyazaki, Yasumitsu Kondoh, Makoto Muroi, Nobumoto Watanabe, Hiroyuki Osada, Ryoung Shin

**Affiliations:** 1 Environmental Response Research Unit, RIKEN Center for Sustainable Resource Science, 1-7-22 Suehirocho, Tsurumi-ku, Yokohama, Kanagawa 230-0045 Japan; 2 Chemical Biology Research Group, RIKEN Center for Sustainable Resource Science, 2-1 Hirosawa, Wako, Saitama 351-0198 Japan; 3 Bioprobe Application Research Unit, RIKEN Center for Sustainable Resource Science, 2-1 Hirosawa, Wako, Saitama 351-0198 Japan; 4 Present Address: Biological Resource Center, Korea Research Institute of Bioscience and Biotechnology, 181 Ipsin-gilJeollabuk-do, Jeongeup, 56212 Korea; 5 Present Address: Galdieria, Co., Ltd., 2-4-2-709 Nihonbashi Hama-cho, Chuo-ku, Tokyo, 103-0007 Japan

**Keywords:** Environmental sciences, Plant sciences, Plant biotechnology, Plant molecular biology, Plant physiology, Plant stress responses

## Abstract

Cesium (Cs) is found at low levels in nature but does not confer any known benefit to plants. Cs and K compete in cells due to the chemical similarity of Cs to potassium (K), and can induce K deficiency in cells. In previous studies, we identified chemicals that increase Cs tolerance in plants. Among them, a small chemical compound (C_17_H_19_F_3_N_2_O_2_), named CsToAcE1, was confirmed to enhance Cs tolerance while increasing Cs accumulation in plants. Treatment of plants with CsToAcE1 resulted in greater Cs and K accumulation and also alleviated Cs-induced growth retardation in *Arabidopsis*. In the present study, potential target proteins of CsToAcE1 were isolated from *Arabidopsis* to determine the mechanism by which CsToAcE1 alleviates Cs stress, while enhancing Cs accumulation. Our analysis identified one of the interacting target proteins of CsToAcE1 to be BETA-GLUCOSIDASE 23 (AtβGLU23). Interestingly, *Arabidopsis atβglu23* mutants exhibited enhanced tolerance to Cs stress but did not respond to the application of CsToAcE1. Notably, application of CsToAcE1 resulted in a reduction of Cs-induced *AtβGLU23* expression in wild-type plants, while this was not observed in a high affinity transporter mutant, *athak5*. Our data indicate that AtβGLU23 regulates plant response to Cs stress and that CsToAcE1 enhances Cs tolerance by repressing AtβGLU23. In addition, AtHAK5 also appears to be involved in this response.

## Introduction

Cesium (Cs) has similar physical and chemical properties to potassium (K)^1,2^. Although natural environments do not contain large amounts of Cs, high levels of Cs are toxic and have a negative impact on plant growth^3,4^. Exposure to high levels of Cs also causes diseases in humans, such as acute cardiac arrest and hypokalemia. This is because Cs ions can accumulate and function as an analogue of K ion by entering plant cells through the K transport system, consequently resulting in K deficiency in organisms^1,2,4–8^. Cs can also inactivate proteins by interacting with potassium-binding sites, as well as alter gene expression and microRNA processing^3,4,9–12^. We have reported that Cs stress alters a large set of metabolites in plants, including several amino acids, and especially cysteine levels^13^. Notably, our previous study also demonstrated that application of cysteine increases Cs accumulation in plants. The mechanism regulating how Cs ions enter and accumulates in cells, however, has not been completely elucidated. Qi et al.^14^, using *Arabidopsis thaliana* as a model, previously reported that K ion deficiency induced the expression of HIGH-AFFINITY K TRANSPORTER5 (*AtHAK5*), which is a member of the *Arabidopsis* K UPTAKE PERMEASE (AtKUP) family. It was suggested that AtHAK5 was responsible for Cs uptake and Cs accumulation under low K conditions^14^. We recently reported that AtHAK5 was not involved in Cs accumulation under K sufficient conditions, and instead, other types of cation channels, especially CYCLIC NUCLEOTIDE-GATED CHANNLEs (CNGCs)^5^ were involved. AtKUP1 and AtKUP9, have also been shown to be involved in Cs^+^ transport^15,16^.

Understanding the mechanism and the regulation of Cs entry and plant response to Cs is important as it will facilitate phytoremediation of Cs-contaminated soils. Genetic modification of plants or the application of a chemical that induces Cs-tolerance can be used as strategies to control Cs toxicity in plants. Regarding the latter approach, several chemical compounds that function as Cs-tolerance enhancers (CsTolen) and/or Cs-accumulators were identified in previous studies through the screening of synthetic chemical libraries comprising 20,000 small organic compounds^13,17^. The application of one of the CsTolen chemicals, CsTolen A, interrupted Cs uptake into plants by binding with Cs ions outside of cells, thus, rendering the plants more Cs-tolerant by reducing Cs accumulation^17^. Application of methyl cysteinate, a derivative of a sulfur-containing cysteine, was found to enhance cesium accumulation in treated plants^13^. Sulfur-containing metabolites, such as glutathione, were also found to alleviate Cs stress in *Arabidopsis*^18^.

The long and rod-shaped endoplasmic reticulum (ER) in *Arabidopsis* has been extensively studied^19^. Two types of ER bodies have been identified in *Arabidopsis*; a constitutive ER body and an inducible ER body. The former has been named due to their visible detection in epidermal cells of cotyledons, hypocotyls, and roots of young seedlings of *Arabidopsis*, and contains large amounts of PYK10/BETA-GLUCOSIDASE 23 (AtβGLU23) proteins, while the latter is induced in cells by wounding or methyl jasmonate treatment in rosette leaves and contains AtβGLU18^20,21^. Jasmonic acid is a phytohormone that regulates various physiological processes related to plant growth and development, and plant response to abiotic and biotic stresses, such as wound response and insect/pathogen attacks^22–24^. Exogenous application of methyl jasmonate induces the formation of ER bodies, suggesting that ER bodies mediate plant defense response to biotic and abiotic stress. The biogenesis of constitutive ER bodies is controlled by the NAI1.2 transcription factor^25^. The biogenesis of inducible ER bodies, however, is dependent on unidentified transcription factors that downregulate the expression of *TONSOKU-ASSOCIATING PROTEIN 1* (*TSA1*), a *NAI2* homolog, and *AtβGLU18*, which is located downstream of the jasmonic acid signaling pathway^26^. βGLU proteins, as well as ER bodies, have been proposed to play an integral role in plant immunity^27–29^. The expression levels of *TSA1* and *AtβGLU18* were reported to be significantly reduced in the jasmonic acid biosynthesis mutant *aos* (*allene oxide synthase*) and the jasmonic acid-insensitive receptor mutant *coi1* (*coronatine insensitive1*)^30–32^. In a previous study using *aos* and *coi1* mutant plants, we reported that jasmonate biosynthesis and signaling are involved in Cs response^33^. Cs treatment increases the levels of jasmonates^18^ and methyl jasmonate suppresses Cs-induced expression of *AtHAK5*^18^.

In the present study, a chemical which enhances Cs tolerance in plants was found to bind to AtβGLU23. Furthermore, the role of AtβGLU23 in Cs-stress response was investigated, as well as its relation to other previously mentioned factors, such as *AtHAK5* induction and the jasmonic acid dependent pathway, during Cs stress *in planta*.

## Materials and methods

### Plant material and growth conditions

*Arabidopsis thaliana* L. (Heynh) ecotype Columbia-0 (Col-0) and mutants obtained from the Arabidopsis Biological Resource Center (ABRC) (https://abrc.osu.edu/) or those previously described were used; *pyk10-1* (CS69080, *atβglu23*-1)^34,35^, *leb-2* (CS69081, *atβglu23*-2), *athak5-*2 (Salk_005604)^36^, jasmonic acid biosynthesis mutant *aos* (CS6149)^31^, and a jasmonic acid-insensitive mutant *coi1*-16^32^. All plants were grown in the controlled growth facility (16 h light/8 h dark cycles, 23 °C). All of the experiments were conducted with seedlings grown for 8 days on media containing 0.5 mM KCl, 50 μM H_3_BO_3_, 10 μM MnCl_2_, 2 μM ZnSO_4_, 1.5 μM CuSO_4_, 0.075 μM NH_4_Mo_7_O_24_, 74 μM Fe-EDTA, 0.5 mM phosphoric acid, 2 mM Ca(NO_3_)_2_, 0.75 mM MgSO_4_, pH 5.8 with Ca(OH)_2_, 1% (w/v) sucrose, and 1% (w/v) SeaKem agarose (Lonza, Basel, Switzerland) supplemented with or without 0.3 mM CsCl. All treatments were applied directly at seed germination. Experimental research on plants including the collection of plant material was performed in accordance with relevant institutional, national, and international guidelines and legislation.

### Phenotype quantification

The fresh weight of aerial plant parts was determined (n > 60). Primary root lengths were measured in digital images of primary roots using ImageJ (n > 60) software^37^, and statistical differences between sample means were evaluated with a one-way ANOVA followed by a Bonferroni’s multiple comparisons test using Prism software version 5 (GraphPad Software, San Diego, USA).

### Identification of direct targets of a small molecule (CsToAcE1)

Photo-cross-linking of CsToAcE1 with agarose beads, CsToAcE1-PALC (photoaffinity-linker-coated) agarose beads, was conducted as previously described^38^. Eleven-day-old *Arabidopsis* seedlings were sampled and total proteins were isolated in an extraction buffer [50 mM Tri-HCl pH 7.6, 100 mM NaCl, 1 mM EDTA, 100 mM MgCl_2,_ 1% Triton-X, anti-protease (Roche, Basel, Switzerland)]. The extracted proteins were incubated with the chemical-linked or non-linked control beads in the presence of 10 mM CsCl for 18–24 h at 4 °C at a low rotary speed. The incubated beads were precipitated by centrifugation at 4000 rpm, 4 °C for 1 min, and were washed with the extraction buffer on ice. The proteins bound to the beads were eluted in 1 × Laemmli sample buffer at 25 °C for 30 min and were then boiled for 5 min. The eluates were separated in a 10% SDS − PAGE gel, and the bands were visualized using Coomassie blue staining. Selected protein bands were excised from the SDS − PAGE gel and subjected to modified in-gel trypsin digestion using sequencing-grade trypsin (Promega, Wisconsin-Madison, USA). The digestion mixture was separated on a nanoflow LC instrument (Easy nLC) (Thermo Fisher Scientific, Waltham, USA) using a nanoelectrospray ionization spray column (NTCC analytical column, C18, φ75 µm × 100 mm, 3 µm; Nikkyo Technos Co., Tokyo, Japan) coupled to a Q-Exactive mass spectrometer (Thermo Fisher Scientific) equipped with a nanospray ion source. MS and MS/MS data were acquired using the data-dependent top 5 method. The resulting MS/MS data were searched using Mascot search (Matrix Science, London, UK) with the following parameters: Gln → pyro-Glu (N-term Q), oxidation (M), carbamido-methyl (C), and Hex (W)^39^.

### Elemental analysis

Three biological replicates of whole, 8-day-old plantlets (40–60 seedlings pooled per replicate) were collected, rinsed in Milli-Q water, and dried in an oven at 65 °C for 3–4 days. Subsequently, 2 ± 0.1 mg of dried sample was weighed and extracted as described in our previous study^17^. The concentrations of K and Cs were determined with a flame atomic absorption spectrometer AAnalyst 200 (PerkinElmer) or by inductively coupled plasma mass spectrometry (NexION® 300 ICP-MS System, Perkin Elmer, Waltham, USA). The concentrations of K and Cs were calculated based on standard curves generated for each element. Statistical differences between sample means were evaluated with a one-way ANOVA followed by a Bonferroni’s multiple comparisons test using Prism software version 5 (GraphPad Software, San Diego, USA).

### Reverse transcription quantitative PCR (RT-qPCR)

Total RNA was extracted from samples using Trizol (Thermo Fisher Scientific), and cDNA synthesis was performed with MMLV reverse transcriptase (Invitrogen, Carlsbad, USA) and oligo(dT), according to the manufacturer’s instructions. RT-qPCR was performed in a Max3000P qPCR system (Agilent Technologies, Santa Clara, USA) using THUNDERBIRD SYBR qPCR mix (TOYOBO, Osaka, Japan). PCR conditions were as follows: first denaturation at 95 °C for 1 min; 40 cycles of denaturation at 95 °C for 15 s, followed by annealing at 60 °C for 30 s; denaturation at 95 °C for 1 min; and dissociation for a melting curve. RT-qPCR assays utilized three biological replicates for each sample. The primers used were as follows: *AtβGLU23* (forward, 5′-CAATGAGCCATGGGTTTTCT-3′ and reverse, 5′-CGTATCCTGATCGTCCGTCT-3′), *AtHAK5* (forward, 5′-GAGACGGACAAA GAAGAGGAACC-3′ and reverse 5′-CACGACCC TTCCCGACCTAATCT-3′)^40^, and *β-tubulin2* (*TUB2*) (forward 5’-GCCAATCCGGTGCTGGTAACA and reverse 5′-CATACCAGATCCAGTTC CTCCTCCC-3′), the latter of which was used as a reference gene^40^. A Tukey’s comparisons test and student t-test were performed using Prism to determine the statistical significance relative to a control (K).

### β-glucosidase activity assay.

One hundred mg of 8-day-old Arabidopsis seedlings were homogenized by grinding the sample in 0.5 ml of 50 mM sodium phosphate buffer (pH7.0). The homogenate was filtered through a 5.0 µm hydrophilic, polyvinylidene difluoride membrane (Millipore Co., Burlington, USA)^41^. The concentration of protein in each filtered, total extract was determined using a Bradford protein assay (Bio-Rad, Hercules, USA). β-glucosidase activity was assessed using a β-glucosidase assay kit (Abcam, Waltham, USA) following the manufacturer’s protocol. Briefly, 20 µl of filtered total extract was mixed with the working reagent (200 µl of assay buffer with 8 µl of 4-nitrophenyl-β-d-glucopyranoside (β-NPG) substrate) and incubated at 37 °C for 6 h. β-glucosidase activity was monitored as the change in OD_420_ every hour using a spectrophotometer (Beckmann, Oklahoma City, USA). One unit of enzyme was calculated based on catalysing the hydrolysis of 1 µmole of β-NPG per min at pH7.0. The assay was repeated three times and one representative data is presented.

## Results

### Identification of CsToAcE1 cellular target proteins

**Figure 1:**
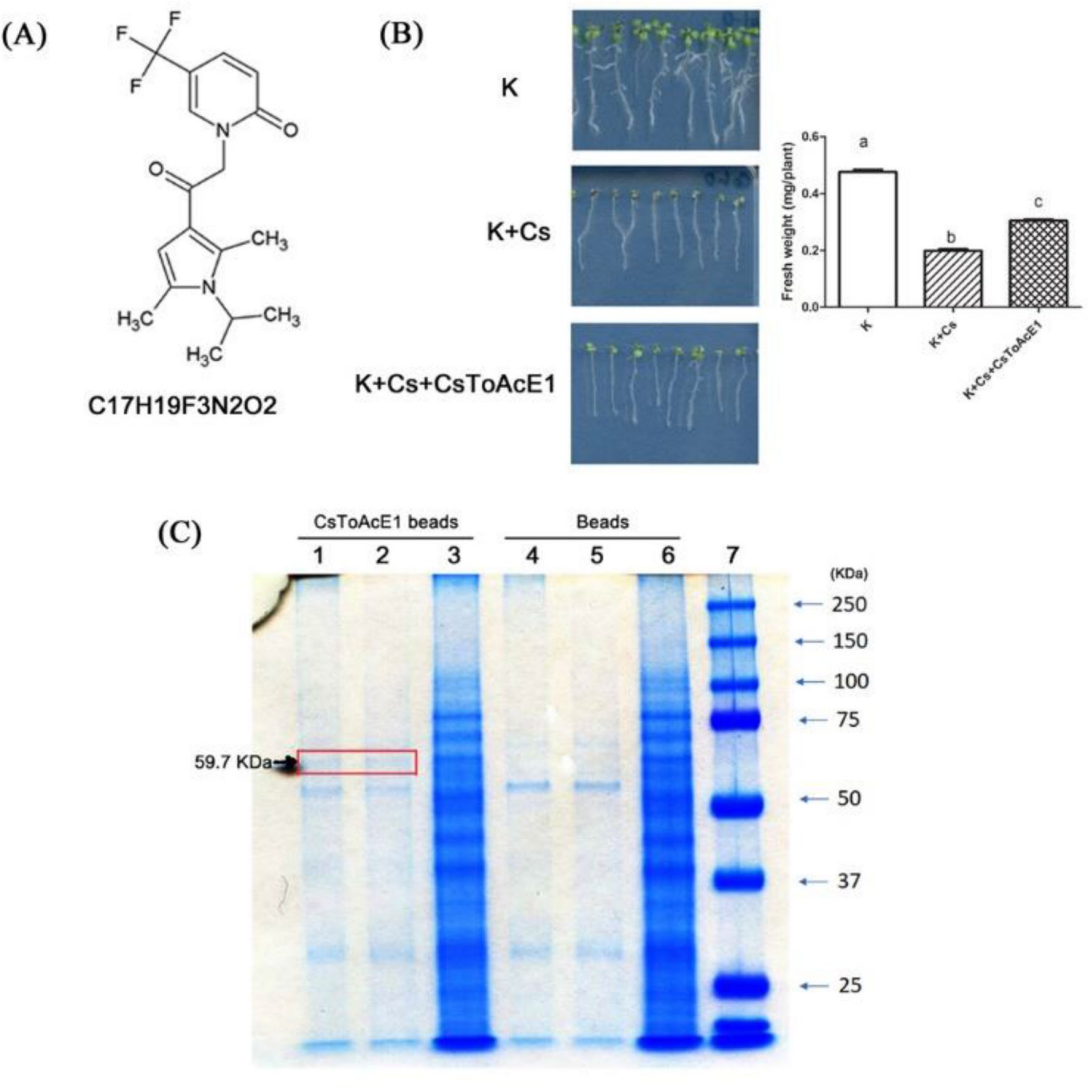
Identification of a CsToAcE1-binding protein in *Arabidopsis*. (**A**) Chemical structure of CsToAcE1 (1-[2-[2,5-Dimethyl-1-(propan-2-yl)-1*H*-pyrrol-3-yl]-2-oxoethyl]-5-(trifluoromethyl)-1,2-dihydropyridin-2-one). (**B**) Phenotype of *Arabidopsis* plants under the condition of sufficient K and Cs stress (0.5 mM K, 0.3 mM Cs), with or without supplementation of the growth medium with 25 µM of CsToAcE1. The fresh weight of seedlings were measured (n > 60). Different letters indicate significant differences (*p* < 0.05) between treatments determined by a one-way ANOVA followed by a Bonferroni’s multiple comparisons test. (**C**) Coomassie blue stained SDS-PAGE gel of proteins eluted from CsToAcE1-PALC agarose beads. Total protein extracts of 11-day-old *Arabidopsis* plants were incubated with either CsToAcE1-PALC agarose beads (CsToAcE1 beads) or control beads without the chemical (Beads). Bound proteins were eluted from the beads at 25 °C (lane 1, 4) or at 100 °C (lane 2, 5). Inputs (lane 3, 6). Size marker (lane 7). The bands present in lanes 1 and 2 and not in lanes 4 and 5 (red box) were excised and subjected to sequence analysis.

Several novel chemical compounds were identified as Cs-stress tolerance-inducing or Cs-accumulation enhancing chemicals in our previous chemical screening analysis^13,17^. Among the chemicals identified in the previous study, C_17_H_19_F_3_N_2_O_2_ (1-[2-[2,5-Dimethyl-1-(propan-2-yl)-1*H*-pyrrol-3-yl]-2-oxoethyl]-5-(trifluoromethyl)-1,2-dihyd ropyridin-2-one) was classified as a Cs-Tolerance Inducer and Cs-Accumulation Enhancer, and designated as CsToAcE1 (Fig. 1A). A modified Hoagland plant media was used to adjust the concentration of K and Cs in the medium. Treatment of *Arabidopsis* plants with 0.3 mM CsCl (K + Cs) triggered growth retardation, however, the application of CsToAcE1 attenuated the Cs-induced growth retardation (Fig. 1B).

In the present study, CsToAcE1-binding target proteins were isolated to elucidate how CsToAcE1 functions in plant cells to improve Cs tolerance, and enhance the accumulation of Cs under Cs stress conditions, at the same time. A pull-down assay using total protein extracts from *Arabidopsis* plants grown on media supplemented with 0.3 mM CsCl recovered a band around 60 kDa (red box), which was only visible in the eluates derived from the CsToAcE1-beads (Fig. 1C, lane 1 and 2). These bands were excised and subjected to peptide sequencing analysis to identify the proteins^38^. A protein extracted from these bands (red box in lane 1 and lane 2) was identified as *Arabidopsis thaliana* BETA-GLUCOSIDASE 23 (AtβGLU23, At3g09260, gi|15232626, UniProtKB-Q9SR37, https://www.uniprot.org/uniprot/Q9SR3 7) with 35.3% sequence coverage and a score of 1057 (Fig. S1).

### AtβGLU23 is involved in plant response to Cs stress

**Figure 2:**
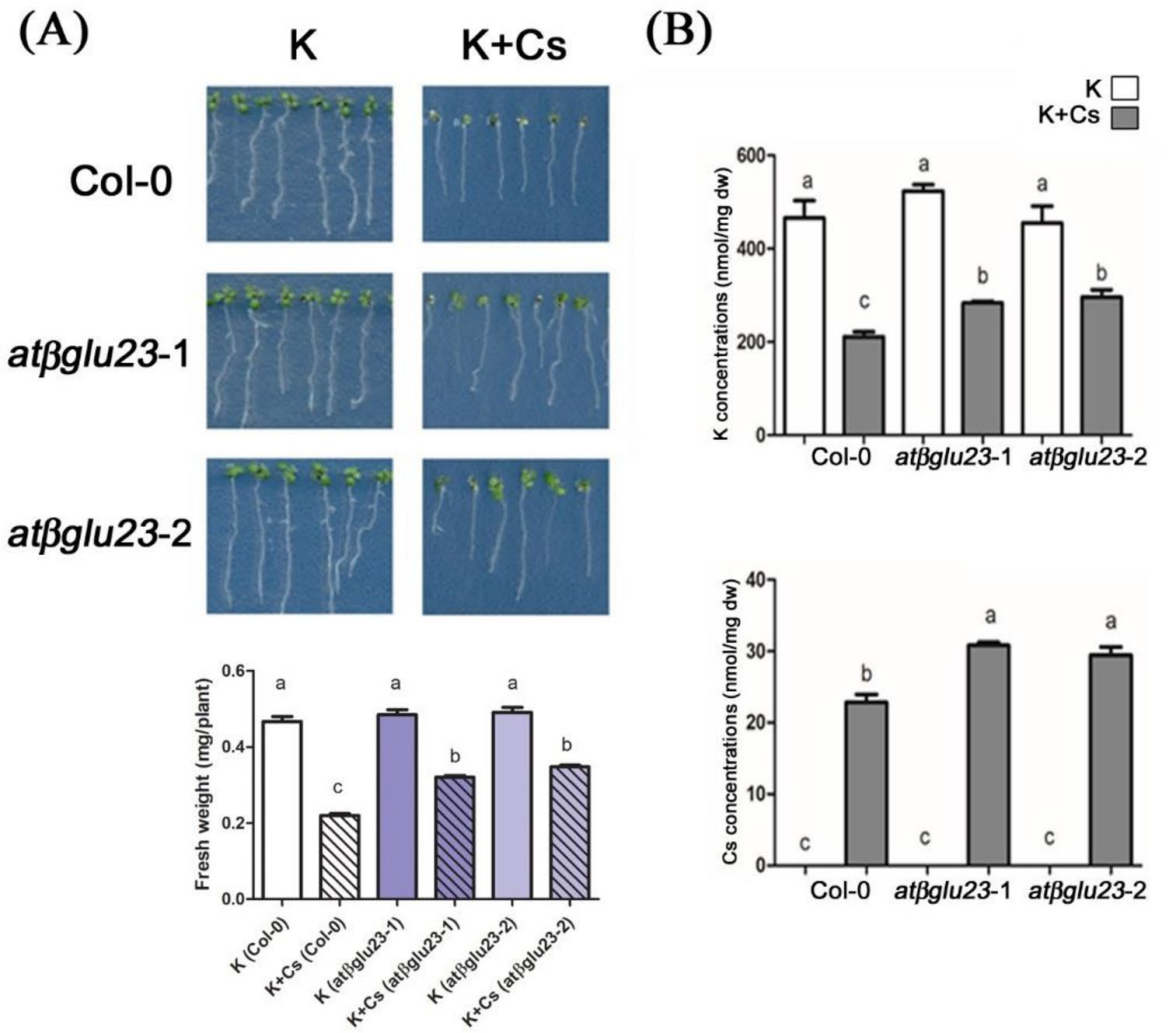
Response of wild-type (Col-0) and *atβglu23* mutant plants to Cs stress. (**A**) Phenotype of wild-type (Col-0), *atβglu23*-1 (*pyk10-*1), and *atβglu23*-2 (*leb-1*) mutants grown under Cs stress and non-stress conditions. Eight-day-old seedlings were grown on agar media containing a suboptimal level of K (0.5 mM KCl), with or without the addition of 0.3 mM CsCl (K + Cs). The fresh weight of seedlings were measured (n > 60). Different letters indicate significant differences (*p* < 0.05) between treatments determined by a one-way ANOVA followed by a Bonferroni’s multiple comparisons test. (**B**) Elemental analysis of K and Cs concentrations *in planta*. K and Cs levels were quantified using AAnalyst. Data represent the mean ± SE (n = 3). Different letters indicate significant differences (*p* < 0.05) between treatments determined by a one-way ANOVA followed by a Bonferroni’s multiple comparisons test.

Two mutant lines of *AtβGLU23* were used to assess the role of CsToAcE1-binding AtβGLU23 protein in Cs uptake and plant response to Cs stress. One of the mutants, originally named *pyk10-1* (CS69080), contains a T-DNA insertion in close proximity to the first exon of *AtβGLU23* (*PYK10-1*) and no transcript is detected in the mutant^33,34^. Another mutant, named *leb*-2, contains a single nucleotide change (CCT → TCT) in the first exon and this change results in an amino acid substitution (P41S) on PYK10 (Dr. Ikuko Hara-Nishimura, personal communication). Transcripts of AtβGLU23 were detected in the *leb*-2 mutant, however, the amount and size of AtβGLU23 protein were altered, similar to the *leb-1* mutant which is a single amino acid change mutant and (C29Y) had the fewer and larger ER bodies^35^. In the present study, the two mutants were designated as *atβglu23-*1 (*pyk10-1*) and *atβglu23-*2 (*leb-2*). The growth of 8-day old Col-0 and *atβglu23* mutant plants in response to 0.3 mM CsCl was compared (Fig. 2). Both *atβglu23*-1 and *atβglu23*-2 mutants exhibited less Cs-induced growth retardation and milder aerial chlorosis relative to the negative effects observed in wild-type plants (Fig. 2A). The alleviation of Cs-induced growth retardation in the mutants suggested the possibility that AtβGLU23 was involved in the response of *Arabidopsis* plants to Cs stress, even in the absence of the application of CsToAcE1. Therefore, Cs and K levels were measured in wild-type and mutant plants utilizing an atomic absorption spectrophotometer to determine if the phenotype of the *atβglu23* mutants and wild-type plants was associated with alterations in the concentration of K or Cs (Fig. 2B). K and Cs levels in wild-type, Col-0 and *atβglu23* mutants were comparable in the absence of the Cs treatment. Notably, Cs and K accumulation in the *atβglu23* mutants was higher than the level of accumulations observed in Col-0 plants in the presence of Cs. These observations suggest that the lack of intact AtβGLU23 conferred tolerance to Cs stress in *Arabidopsis* plants, while accumulating higher levels of Cs, as well as K. The data also indicate that AtβGLU23 is associated with plant response to Cs stress by altering K and Cs accumulation.

### Effect of CsToAcE1 on Arabidopsis plants is analogous to of the effect of AtβGLU23 mutation

**Figure 3:**
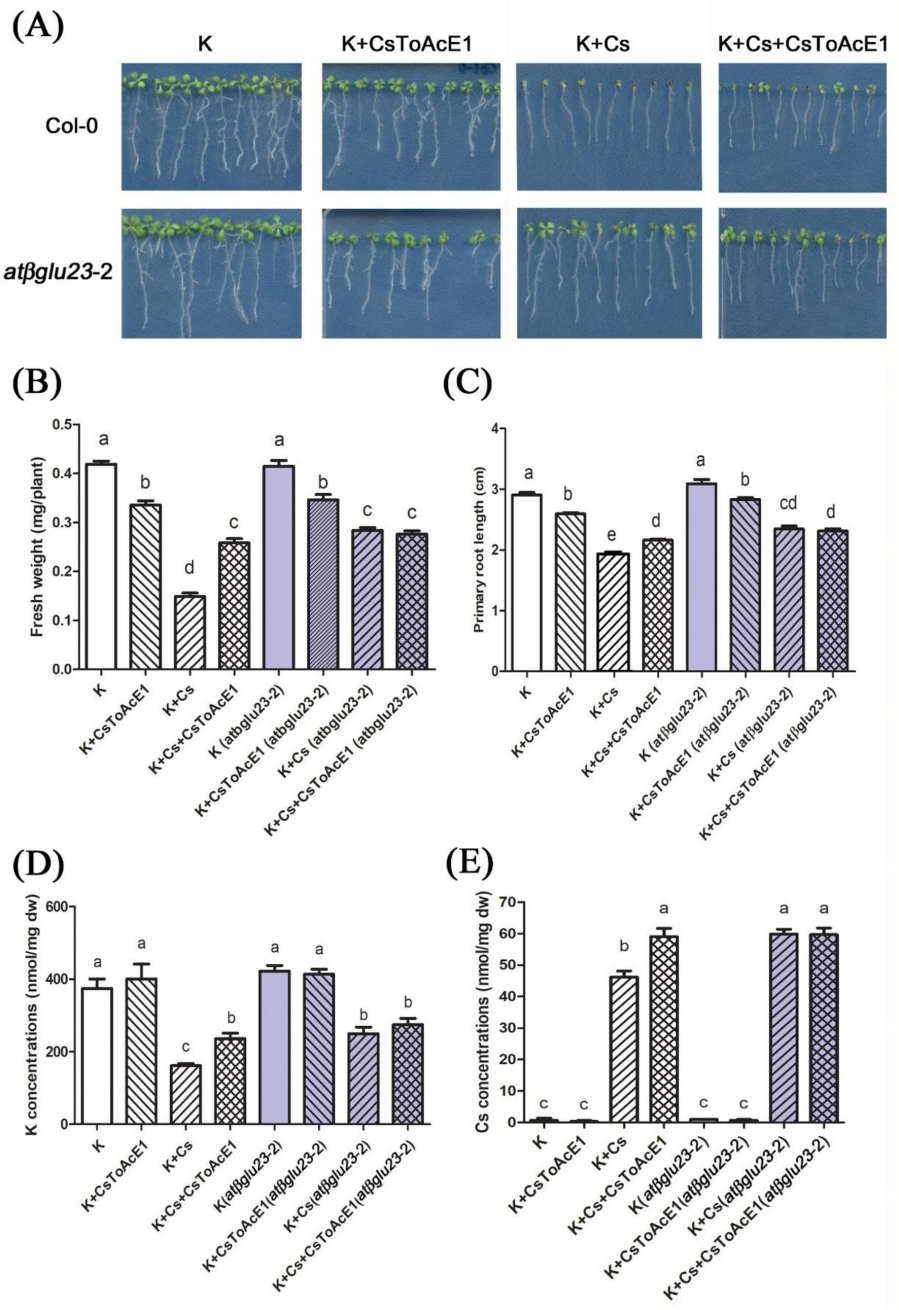
Effect of CsToAcE1 on Cs stressed wild-type (Col-0) and *atβglu23* mutant *Arabidopsis* plants. (**A**) Response of wild-type and *atβglu23-*2 mutant *Arabidopsis* plants to CsToAcE1 under Cs-stress (K plus Cs plus CsToAcE1) and non-stress (K plus CsToAcE1) conditions. Plants were grown for 8 days on agar media containing 0.5 mM KCl as a control (K) and media containing 0.3 mM CsCl (+ Cs) with or without the addition of 25 µM CsToAcE1 (+ CsToAcE1). The fresh weight of aerial parts (**B**) and primary root lengths (**C**) were analyzed (n > 60). Elemental analysis of K (**D**) and Cs (**E**) concentrations *in planta*. K and Cs levels were quantified using a NexION 300 ICP-MS System. Data represent the mean ± SE (n = 3). Different letters indicate significant differences (*p* < 0.05) between treatments determined by a one-way ANOVA followed by a Bonferroni’s multiple comparisons test.

Col-0 and the *atβglu23-*2 mutant plants were grown on media supplemented with 25 µl of CsToAcE1, with or without the addition of Cs, to determine if CsToAcE1 is involved in Cs accumulation and tolerance response via AtβGLU23. Supplementation of the medium with CsToAcE1 improved Cs tolerance in Col-0 plant grown under Cs stress, however, *atβglu23-*2 mutant plants were even less susceptible to Cs stress than Col-0 plants and did not exhibit any further enhancement of Cs tolerance in response to the CsToAcE1 treatment (Fig. 3A). Treatment with CsToAcE1 under Cs stress condition resulted in lower reduction in the fresh weight and root growth relative to the untreated control in the wild-type plants, (Fig. 3B,C), while the CsToAcE1 treatment had no effect on the growth of the Cs-stressed *atβglu23-*2 mutant plants (Fig. 3B,C). Elemental analysis revealed that the mutation in the *AtβGLU23* gene resulted in higher K accumulation under Cs-stress conditions, which was consistent with our initial data (Figs. 2B and 3D). Interestingly, the CsToAcE1 treatment resulted in improved K accumulation in Col-0 plants exposed to Cs stress to a level that was equivalent to that of *atβglu23-*2 plants, while the K content in the *atβglu23-*2 mutant was not further enhanced by the CsToAcE1 treatment (Fig. 3D). Cs content in Col-0 plants increased in response to the CsToAcE1 treatment, however, CsToAcE1 treatment of the *atβglu23-*2 mutant did not induce any further increase in Cs levels beyond the elevated Cs levels observed in the absence of the CsToAcE1 treatment (Fig. 3E). No synergetic or antagonistic effect of the CsToAcE1 treatment was observed in the response of *atβglu23-*2 mutant plants to Cs stress. The determination that CsToAcE1 does not further enhance Cs tolerance or Cs accumulation in the absence of AtβGLU23 in the *atβglu23-*2 mutant suggests that AtβGLU23 is the target protein of CsToAcE1.

### CsToAcE1 regulates AtβGLU23 and AtHAK5 expression

**Figure 4:**
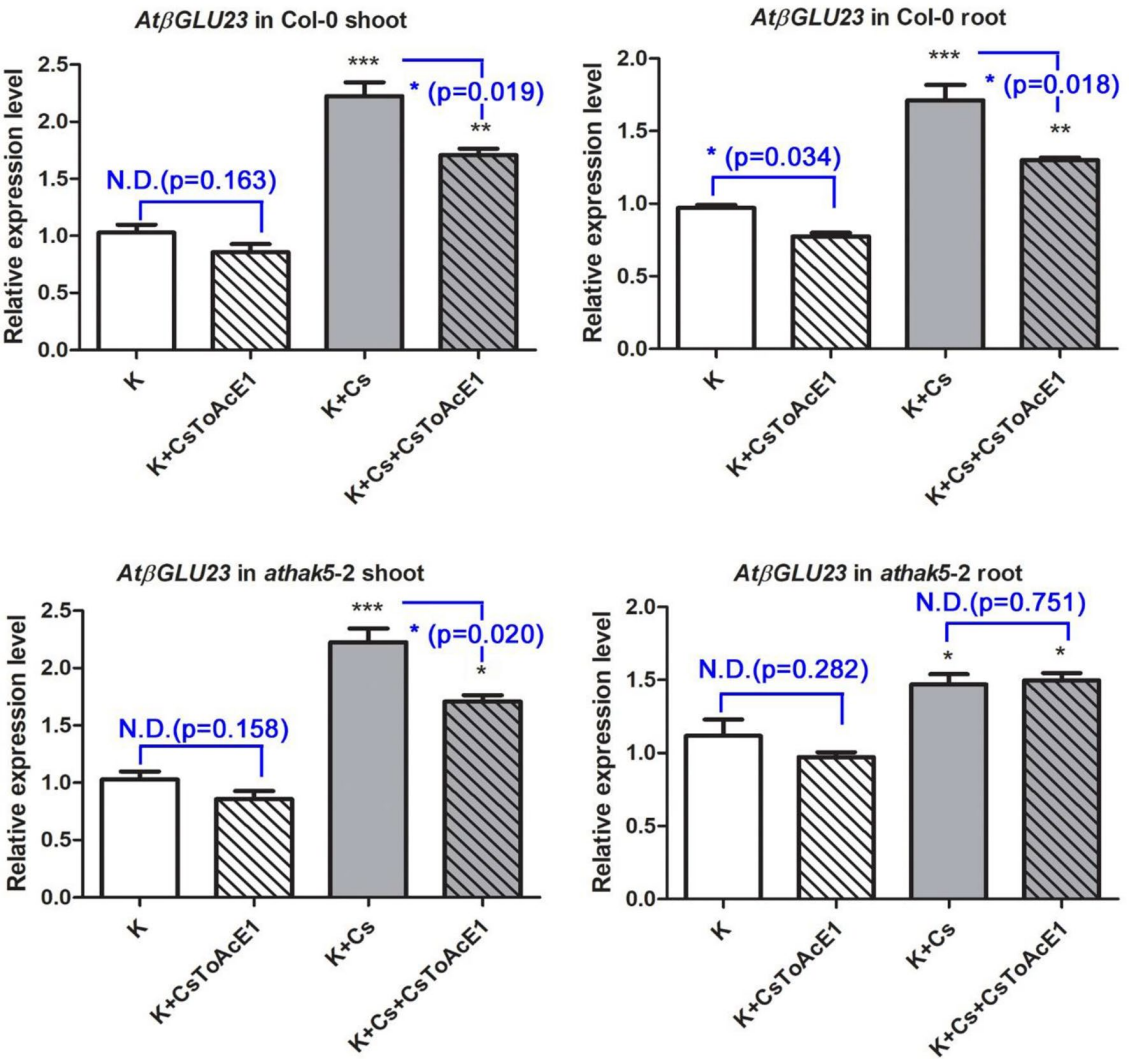
Analysis of *AtβGLU23* expression. The relative expression of *AtβGLU23* determined in 8-day-old Col-0 and *athak5*-2 shoot and root samples, in response to 25 µM CsToAcE1, Cs, or a combination of Cs and CsToAcE1. Error bars indicate standard errors (n = 3). Statistical differences relative to a control (K) were determined using a Tukey’s comparisons test and statistical differences between no CsToAcE1 treated and CsToAcE1 treated were determined using student t-tests (N.D., no difference; *p < 0.05; **p < 0.01; ***p < 0.001).

Analysis of *AtβGLU23* expression in wild-type Col-0 and mutant plants indicated that *AtβGLU23* expression in both shoots and roots of Col-0 plants increased approximately two-fold in response to the Cs-stress treatment, relative to control plants (no Cs stress). *AtβGLU23* expression in the presence of Cs, however, was attenuated by the CsToAcE1 treatments in Col-0 shoots. *AtβGLU23* expression was also attenuated by the CsToAcE1 treatments regardless of the presence or absence of Cs in Col-0 roots (Fig. 4). In contrast, the *AtβGLU23* expression in *athak5*-2 roots was not attenuated by the CsToAcE1 treatment (Fig. 4). These data indicate that CsToAcE1 has little effect on *AtβGLU23* expression in the *athak5*-2 roots.

**Figure 5:**
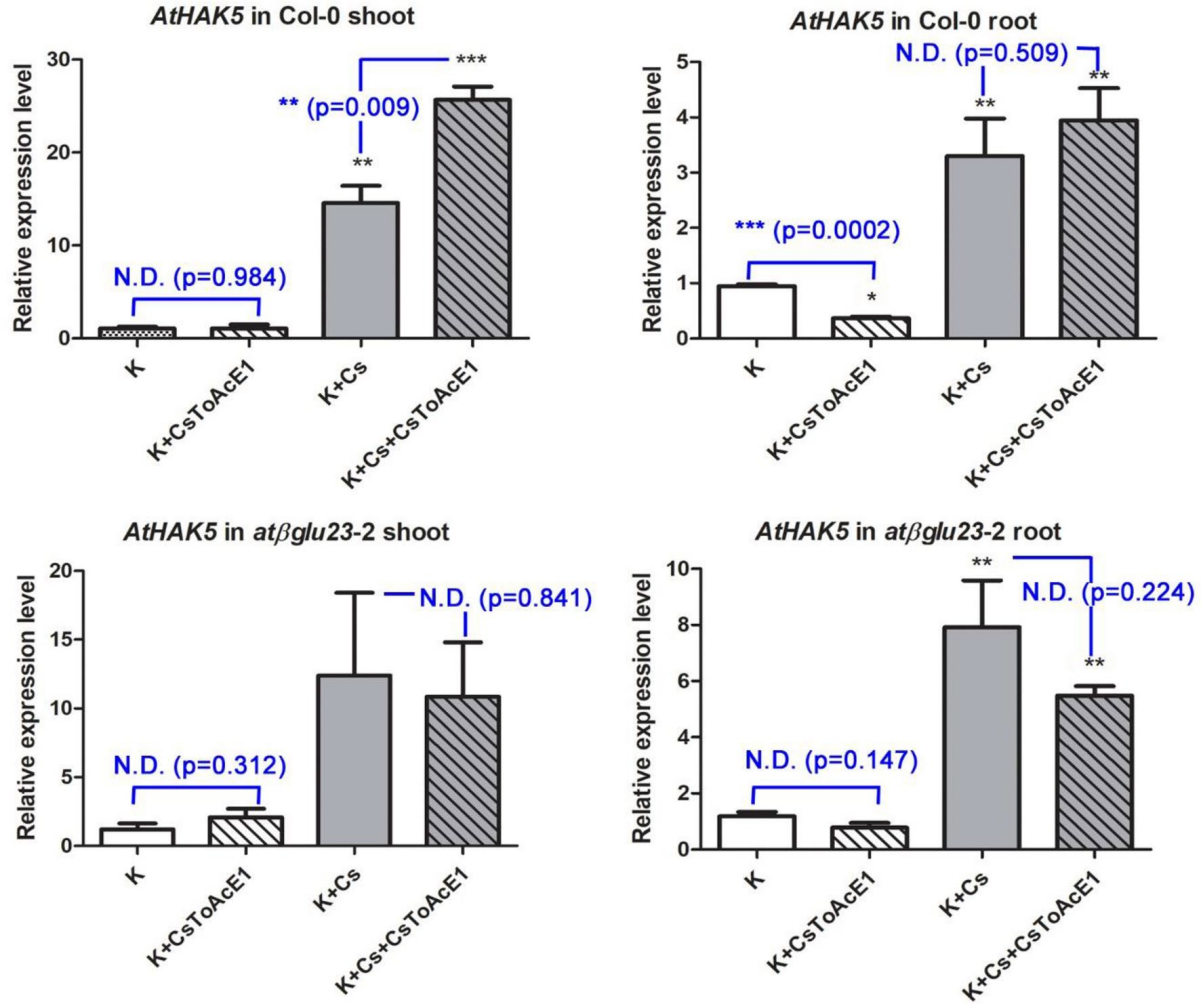
Analysis of *AtHAK5* expression. The relative expression of *AtHAK5* determined in 8-day-old Col-0 shoot and *atβglu23*-2 root samples in response to 25 µM CsToAcE1, Cs, or a combination of Cs and CsToAcE1. The expression of *AtHAK5* was used as a marker for K deficiency *in planta*. Error bars indicate standard errors (n = 3). Statistical differences relative to a control (K) were determined using a one-way ANOVA followed by a Tukey’s comparisons test and statistical differences between no CsToAcE1 treated and CsToAcE1 treated were determined using student t-tests (N.D., no difference; *p < 0.05; **p < 0.01; ***p < 0.001).

The expression of *AtHAK5*, a K-deficiency marker, which was previously shown to be induced by the Cs treatment^5^, was induced by the Cs treatment in Col-0 shoots and roots but only in the roots of *atβglu23-*2 (Fig. 5). However, the expression of *AtHAK5* was not attenuated by CsToAcE1 in both Col-0 and the *atβglu23-*2 roots and was increased by CsToAcE1 in the presence of Cs in Col-0 shoots unlike *AtβGLU23* expression (Figs. 4 and 5). Our previous study demonstrated that a jasmonic acid biosynthesis mutant (*aos*) and a jasmonic acid-insensitive mutant (*coi1*-16) exhibited greater tolerance to Cs stress than wild type plants and that Cs induces jasmonate biosynthesis and signaling^32^. Therefore, the expression of *AtβGLU23* in *aos* and *coi1*-16 was analyzed to determine the involvement of jasmonic acid signaling in *AtβGLU23* expression (Fig. S2). Results indicated that *AtβGLU23* expression was upregulated in *aos* and *coi1-*16 mutants in response to the Cs treatment, which was similar to its expression in Col-0 plants. Unlike Col-0, however, the increased *AtβGLU23* expressions by Cs treatments were not attenuated by CsToAcE1 in roots of *athak5*-2 mutant and the jasmonate-related mutants (Fig. S2 and Fig. 4).

### CsToAcE1 negatively regulates β-glucosidase activity

**Figure 6:**
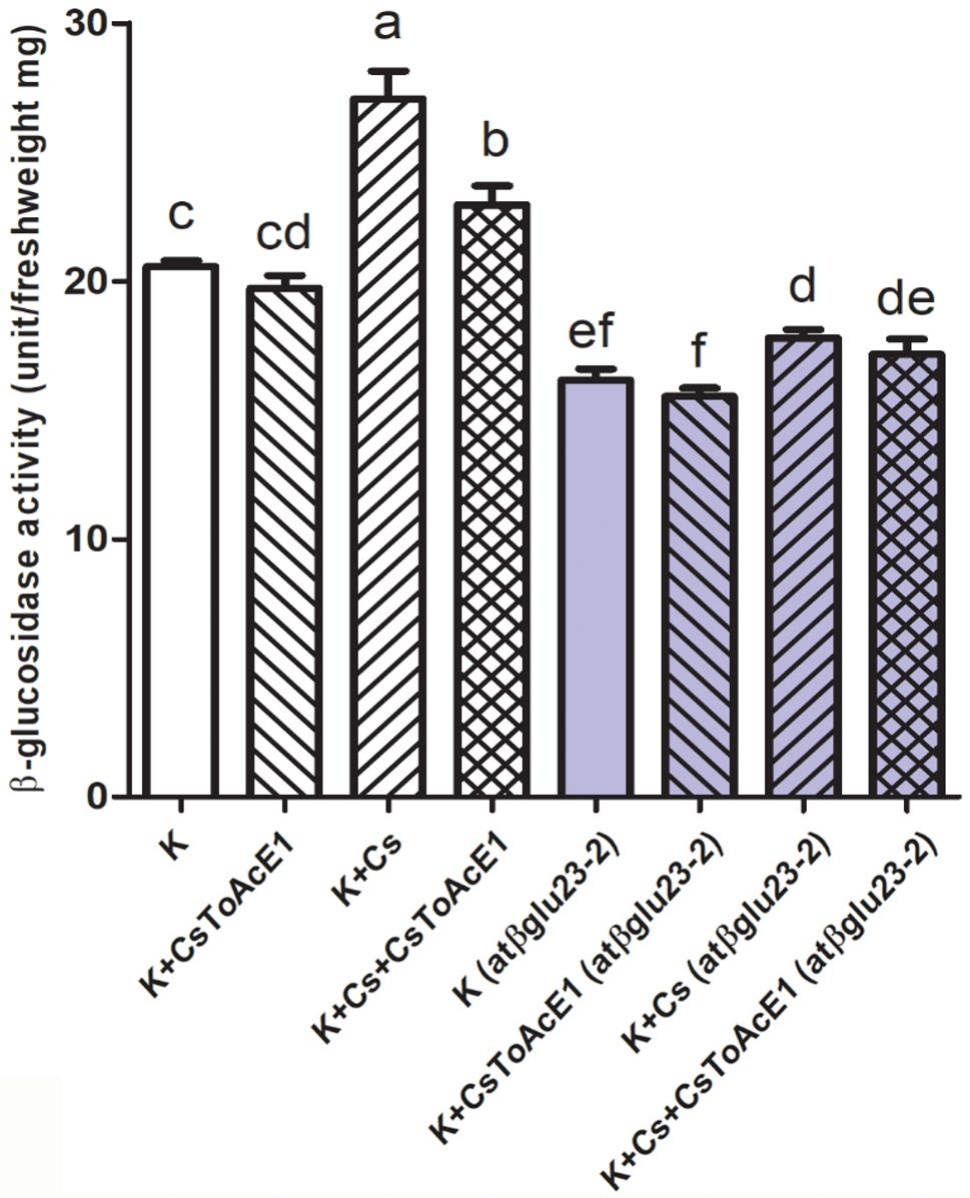
Analysis of β-glucosidase activity. The β-glucosidase activity of wild-type and *atβglu23-*2 mutant *Arabidopsis* plants to CsToAcE1 under Cs-stress (K plus Cs plus CsToAcE1) and non-stress (K plus CsToAcE1) conditions. Plants were grown for 8 days on agar media containing 0.5 mM KCl as a control (K) and media containing 0.3 mM CsCl (+ Cs) with or without the addition of 25 µM CsToAcE1 (+ CsToAcE1). *Arabidopsis* plants were grown for 8 days on agar media containing 0.5 mM KCl as a control (K) and on agar media containing 0.3 mM CsCl (+ Cs) with or without the addition of 25 µM CsToAcE1 (+ CsToAcE1). The assay kit utilized 4-nitrophenyl-β-d-glucopyranoside as a substrate. Enzyme activity was assessed by monitoring the change in OD_420_ in a spectrophotometer for 3 h. One unit of enzyme was calculated based on catalysing the hydrolysis of 1 µmole of β -NPG per min at pH7.0. Data represent the mean ± SE (n = 3). Different letters indicate significant differences (*p* < 0.05) between treatments determined by a one-way ANOVA followed by a Bonferroni’s multiple comparisons test.

β-glucosidase activity was measured in wild-type and *atβglu23*-2 plants to better understand how CsToAcE1 is involved in the regulation of AtβGLU23. β-glucosidase activity was induced by Cs-stress, while the CsToAcE1 treatment reduced the Cs-induced β-glucosidase activity in wild-type plants (Fig. 6). Overall enzyme activity was reduced in *atβglu23*-2 plants, consistent with the past data on another *atβglu23* mutant^41^. In contrast to the β-glucosidase activity observed in wild-type plants, the increase in β-glucosidase activity by Cs-stress in *atβglu23*-2 plants was attenuated and hardly any reduction in enzyme activity was observed in *atβglu23*-2 plants in response to the CsToAcE1 treatment (Fig. 6).

**Figure 7:**
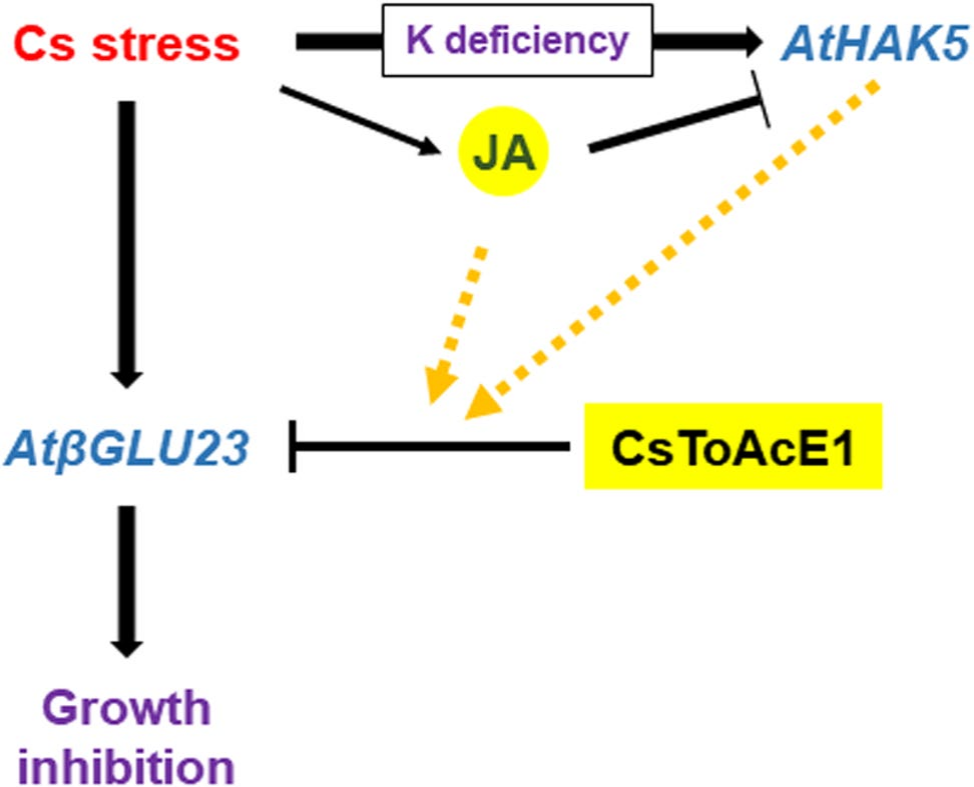
A proposed model for the binding of AtβGLU23 with CsToAcE1 under Cs stress conditions. Blue letters indicate the genes. Arrow bars indicate positive interaction and the orange arrow bars indicate possible positive interactions, respectively. T-signs bars indicate the negative interactions. JA indicates jasmonates.

In summary, Cs stress increases *AtβGLU23* transcript levels and enzyme activity, and induces growth inhibition. A Cs tolerance-enhancing chemical, CsToAcE1, binds to AtβGLU23, which is potentially mediated by jasmonates and AtHAK5, and reduces Cs-induced growth inhibition (Fig. 7).

## Discussion

In the present study, AtβGLU23 in *Arabidopsis* was identified as the target protein of the Cs-tolerance enhancer and Cs-accumulator chemical, CsToAcE1. Interestingly, *atβglu23* mutants exhibited a Cs tolerant phenotype, which was similar to that of wild-type plants treated with CsToAcE1 (Figs. 2 and 3). Reduced levels of AtβGLU23 were associated with a reduction in the growth retardation induced by Cs, and it appears that the level of Cs in mutant plants is not correlated with the level of Cs tolerance (Fig. 3). It is plausible that AtβGLU23 is involved in the response to Cs stress rather than in regulating the uptake of external Cs. CsToAcE1 may moderately suppress the negative effect of AtβGLU23 on plant growth in response to Cs, thus, conferring enhanced Cs tolerance to plants. RT-qPCR data indicate that *AtβGLU23* is induced when Cs is applied and that the induced level of *AtβGLU23* expression is somewhat attenuated by the CsToAcE1 treatment (Fig. 4). Furthermore, an increase in β-glucosidase activity was observed under Cs-stress conditions in wild-type plants but was alleviated by CsToAcE1. Comparatively, the induction of β-glucosidase activity by Cs stress and its alleviation by CsToAcE1 was much less in *atβglu23*-2 plants (Fig. 6). These results support the hypothesis that CsToAcE1 negatively regulates *AtβGLU23* in plants under Cs stress. The non-responsiveness of the *atβglu23* mutants to CsToAcE1 supports the premise that AtβGLU23 is the target of this chemical.

Beta-glucosidases are known to be the main components of ER bodies and AtβGLU23 (PYK10) and AtβGLU18 are the most abundant ER body proteins in *Arabidopsis*. AtβGLU21 and AtβGLU22 have also been suggested to regulate constitutive ER body formation, while AtβGLU18 is involved in inducible ER body formation in response to wounding and methyl jasmonate^20,21,34,42^. Several studies have suggested that the ER bodies play a role in plant response to biotic and abiotic stress^20,27–29,43^. Data obtained in our previous study suggests a link between Cs stress and jasmonate synthesis and signaling^33^. In the present study, inhibition of *AtβGLU23* expression by CsToAcE1 in the presence of Cs was not observed in jasmonic acid mutants (Fig. S2), suggesting that jasmonates may play a role in CsToAcE1-AtβGLU23-mediated Cs response. In a previous study, Wang et al. demonstrated that the transcript level of *AtβGLU18* was significantly reduced in *aos* and *coi1*-16, relative to basal levels expressed in wild-type plants^30^. In addition, the exogenous application of methyl jasmonate was found to induce the biogenesis of ER bodies, which in *Arabidopsis* requires AtβGLU protein. An interaction between jasmonic acid and AtβGLU proteins in plant response to various stresses, including Cs, has been reported^20,21^.

In our present study, Cs-induced *AtβGLU23* expression was partly inhibited by CsToAcE1, however, this inhibition was not observed in the *athak5* roots (Fig. 4). In contrast, Cs-induced *AtHAK5* expression was not inhibited by CsToAcE1 in either Col-0 or the *atβglu23*-2 mutant (Fig. 5). AtHAK5 protein has been reported to be primarily located in ER bodies when K is sufficient and localized to the plasma membrane under K-deficient conditions^14^. Therefore, Cs-induced K deficiency in plants may also lead to the translocation of AtHAK5 from ER bodies to the plasma membrane. Furthermore, Cs-induced *AtβGLU23* expression and its downregulation by CsToAcE1 may be linked to the translocation of AtHAK5 due to the K status. Therefore, further studies are warranted to evaluate the effect of CsToAcE1 on the subcellular localization of AtHAK5 and AtβGLU23 in *Arabidopsis* plants. Notably, the overexpression of an *Arabidopsis* beta-glucosidase, *AtBG1*, which hydrolyzes inactive, glucose-conjugated abscisic acid to active abscisic acid, has been demonstrated to enhance tolerance to drought and salt stress^44^. Therefore, AtβGLU proteins appear to be broadly involved in plant response to a variety of stresses. In summary, we demonstrated that AtβGLU23 plays a negative role in Cs stress tolerance and CsToAcE1 attenuates the negative effects of Cs through the inhibition of AtβGLU23 activity (Fig. 7). Further studies detailing the functional mechanisms of the relationship between AtβGLU23, Cs stress, and CsToAcE1 are in progress to increase our understanding of Cs response in plants.

## Supplementary Information

Supplementary Figures.
