# Supplementary material for: Cesium tolerance is enhanced by a chemical which binds to BETA-GLUCOSIDASE 23 in Arabidopsis thaliana
Source: Sci Rep. 2021 Oct 26;11:21109. doi: 10.1038/s41598-021-00564-4 (PMC8548588; doi:10.1038/s41598-021-00564-4)
Supplement: Supplementary file 1 — Supplementary Figures. [file 41598_2021_564_MOESM1_ESM.pdf]

(A)

gi|15232626 Mass: 59683 Score: 1057 Matches: 26(26) Sequences: 14(14)

beta-glucosidase 23 [*Arabidopsis thaliana*]

Observed	Mr expt	Mr calc	Score	Expect	Peptide
696.3792	695.3719	695.3715	30	0.028	K.DIVGHR.L
438.7235	875.4323	875.4323	38	0.013	R.MSIAWPR.I
928.4183	927.4110	927.4120	29	0.035	R.CSSYVNAK.C
464.7132	927.4118	927.4120	36	0.0062	R.CSSYVNAK.C
475.7390	949.4634	949.4617	32	0.048	K.NLNTDAFR.M
576.2926	1150.5706	1150.5699	47	0.0017	R.FGLYYVDFK.N
625.8036	1249.5926	1249.5914	44	0.0028	R.GPALWDIYCR.R
473.2657	1416.7752	1416.7725	33	0.047	K.DFLSQGVRPSALK.K
709.3952	1416.7758	1416.7725	66	2.2e-005	K.DFLSQGVRPSALK.K
770.3633	1538.7120	1538.7075	55	0.00022	R.WMQDSLITWESK.N
776.8527	1551.6909	1551.6882	50	0.00043	R.EYADFVFQEYGGK.V
780.3826	1558.7507	1558.7483	75	2.3e-006	R.HLLSMQEAVCIDK.V
890.3795	1778.7445	1778.7431	121	1.3e-011	R.CNNDNGDVAVDFFHR.Y
593.9224	1778.7453	1778.7431	112	1e-010	R.CNNDNGDVAVDFFHR.Y
751.0666	2250.1779	2250.1756	36	0.025	K.NAQNYAIGSKPLTAALNVYSR.G
902.7902	2705.3487	2705.3449	85	3.8e-007	R.SGYEAYLVTHNLLISHAEAVEAYR.K
927.1081	2778.3024	2778.2998	82	5.6e-007	K.IGIAHSPAWFEAHDLADSQDGASIDR.A

(B)

```
  1 MVLQKLPLIG LLLLLTIVAS PANADGPVCP PSNKLSRASF PEGFLFGTAT
 51 AAYQVEGAIN ETCRGPALWD IYCRRYPERC NNDNGDVAVD FFHRYKEDIQ
101 LMKNLNTDAF RMSIAWPRIF PHGRKEKGVS QAGVQFYHDL IDELIKNGIT
151 PFVTVFHWDT PQDLEDEYGG FLSERIVKDF REYADFVFQE YGGKVKHWIT
201 FNEPWVFSHA GYDVGKKAPG RCSSYVNAKC QDGRSGYEAY LVTHNLLISH
251 AEAVEAYRKC EKCKGGKIGI AHSPAWFEAH DLADSQDGAS IDRALDFILG
301 WHLDTTTFGD YPQIMKDIVG HRLPKFTTEQ KAKLKASTDF VGLNYYTSVF
351 SNHLEKPDPS KPRWMQDSLI TWESKNAQNY AIGSKPLTAA LNVYSRGFRS
401 LLKYIKDKYA NPEIMIMENG YGEELGASDS VAVGTADHNR KYYLQRHLLS
451 MQEAVCIDKV NVTGYFVWSL LDNFEWQDGY KNRFGLYYVD FKNNLTRYEK
501 ESGKYYKDFL SQGVRPSALK KDEL
```

Figure S1. Peptide sequencing analysis of the CsToAcE1-binding protein.

Identification of the CsToAcE1 binding protein. (A) The observed tryptic peptides are based on their position, experimentally determined m/z values, monoisotopic mass (Mr expt), theoretically calculated (Mr calc) values, score (the calculated probability that the observed matches between the experimental data and database data is a random event). (B) Amino acid sequences of AtβGLU23. The representation of sequence coverage by the MS/MS analysis is shown in red.

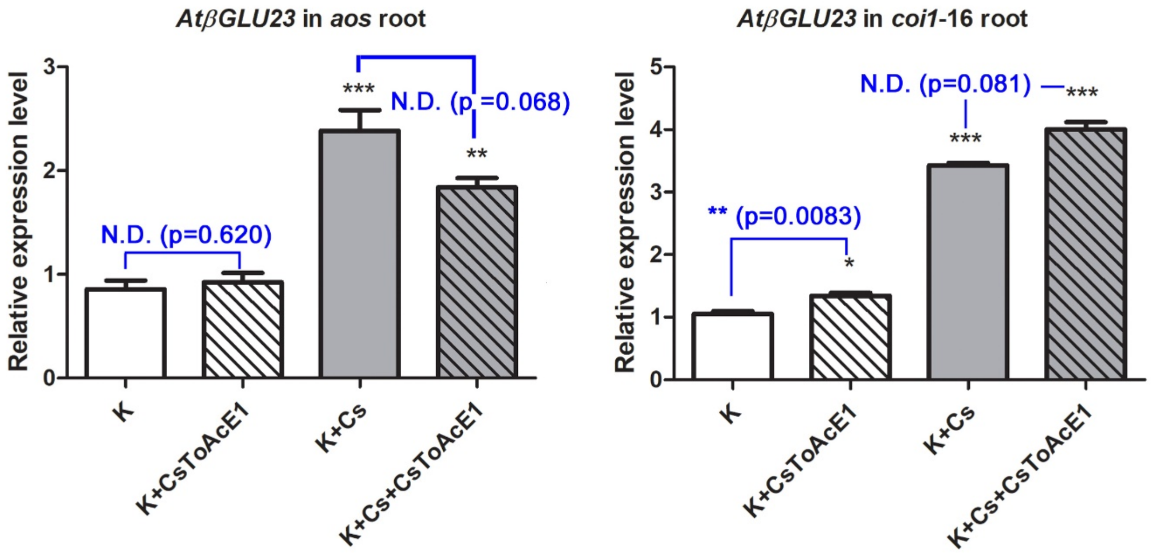

Figure S2. Analysis of *AtβGLU23* expression in roots of *aos* and *coi1* mutants under Cs stress conditions.

Expression of *AtβGLU23* in roots of *aos* (left) and *coi1*-16 (right) mutant plants grown on media with or without the addition of 0.3 mM CsCl, 25 μM CsToAcE1, or a combination of both. Error bars indicate standard errors (n=3). Statistical differences relative to a control (K) were determined using a Tukey's comparisons test (*, $p < 0.05$; **, $p < 0.01$; ***, $p < 0.001$). N.D. indicates no statistical difference.
